# Supplementary material for: Deep mutational scanning of SARS-CoV-2 receptor binding domain reveals constraints on folding and ACE2 binding
Source: bioRxiv. 2020 Jun 17:2020.06.17.157982. Preprint. [Version 1] doi: 10.1101/2020.06.17.157982 (PMC7310626; doi:10.1101/2020.06.17.157982)
Supplement: Supplement 2 [file media-2.html]

xml version="1.0" encoding="UTF-8"?

# SARS-CoV-2 RBD DMS

### Instructions

- Hover over cells with mouse to reveal additional information.
- Select site subsets using the drop down menu below the plots.
- Change which sites are displayed by brushing the zoom bar and dragging the brush.
- Clear the zoom bar by double clicking it.
- Structural visualizations of the data are available via `dms-view` here
- Raw data available on GitHub

'
+ '

JavaScript Error: ' + error.message + '

'
+ "

This usually means there's a typo in your chart specification. "
+ "See the javascript console for the full traceback.

"
+ '

');
throw error;
}
const el = document.getElementById('vis');
vegaEmbed("#vis", spec, embedOpt)
.catch(error => showError(el, error));
})(vegaEmbed);
