# Supplementary material for: Deep mutational scanning of SARS-CoV-2 receptor binding domain reveals constraints on folding and ACE2 binding
Source: bioRxiv. 2020 Jun 17:2020.06.17.157982. Preprint. [Version 1] doi: 10.1101/2020.06.17.157982 (PMC7310626; doi:10.1101/2020.06.17.157982)
Supplement: 1 [file NIHPP2020.06.17.157982-supplement-1.pdf]

bioRxiv preprint doi: https://doi.org/10.1101/2020.06.17.157982. this version posted June 17, 2020. The copyright holder for this preprint (which was not certified by peer review) is the author/funder. It is made available under a CC-BY 4.0 International license.

Figure S1

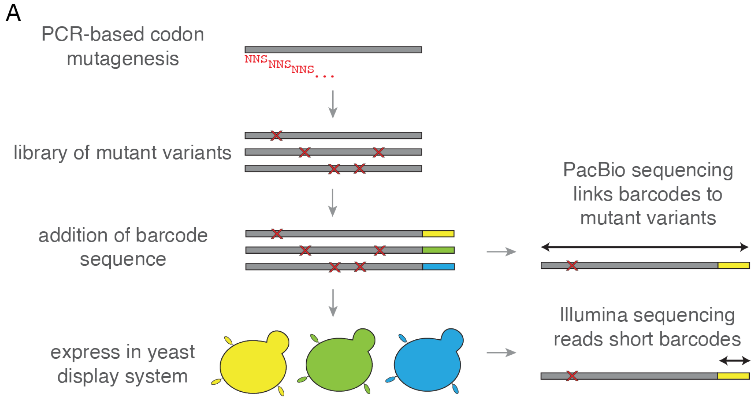

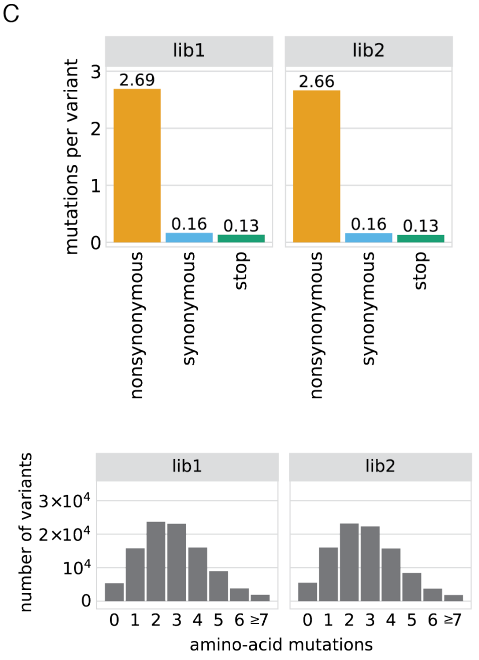

B

library	total number barcodes	average (median) CCS per barcode	fraction barcodes with >1 CCS	accuracy per CCS
1	99,648	3.90 (3)	0.769	0.998
2	97,691	5.11 (4)	0.818	0.998

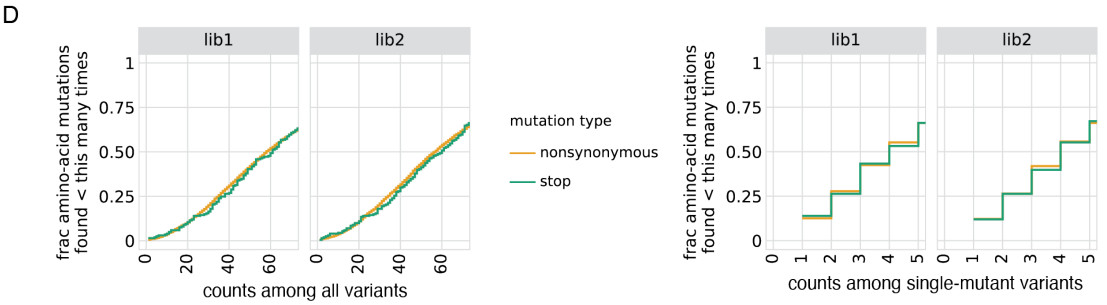

E

library	fraction amino acid mutations in ≥1 variant	fraction amino acid mutations in ≥1 single-mutant variant
1	0.995	0.875
2	0.994	0.878
combined	0.996	0.953

Figure S1. SARS-CoV-2 RBD mutant libraries. (A) Scheme of the library generation and sequencing approach. SARS-CoV-2 RBD mutant libraries were constructed in fully independent duplicates, and variants were linked to barcodes by long-read PacBio sequencing. (B) PacBio sequencing stats on duplicate SARS-CoV-2 mutant libraries. Comparison of RBD sequences among independent circular consensus sequences (CCSs) of the same barcode enables calculation of an empirical accuracy, which describes the minimal expected accuracy of the barcode:RBD linkage for barcodes with a single CCS (see Methods for details). Most barcodes were represented by multiple CCSs, which further increases the accuracy of barcode:RBD linkage. (C) Statistics on mutation rates in mutant libraries. Top, average number of mutations of different types across variants in each library. Bottom, distribution of number of amino-acid mutations per variant. (D, E) Mutation coverage in mutant libraries. Cumulative distribution plots (D) give the fraction of all possible amino-acid mutations observed in the indicated number of variants, including all variants (left) or only variants with a single mutation (right). Minimum coverage statistics from these curves are tabulated in (E).

bioRxiv preprint doi: https://doi.org/10.1101/2020.06.17.157982. this version posted June 17, 2020. The copyright holder for this preprint (which was not certified by peer review) is the author/funder. It is made available under a CC-BY 4.0 International license.

Figure S2

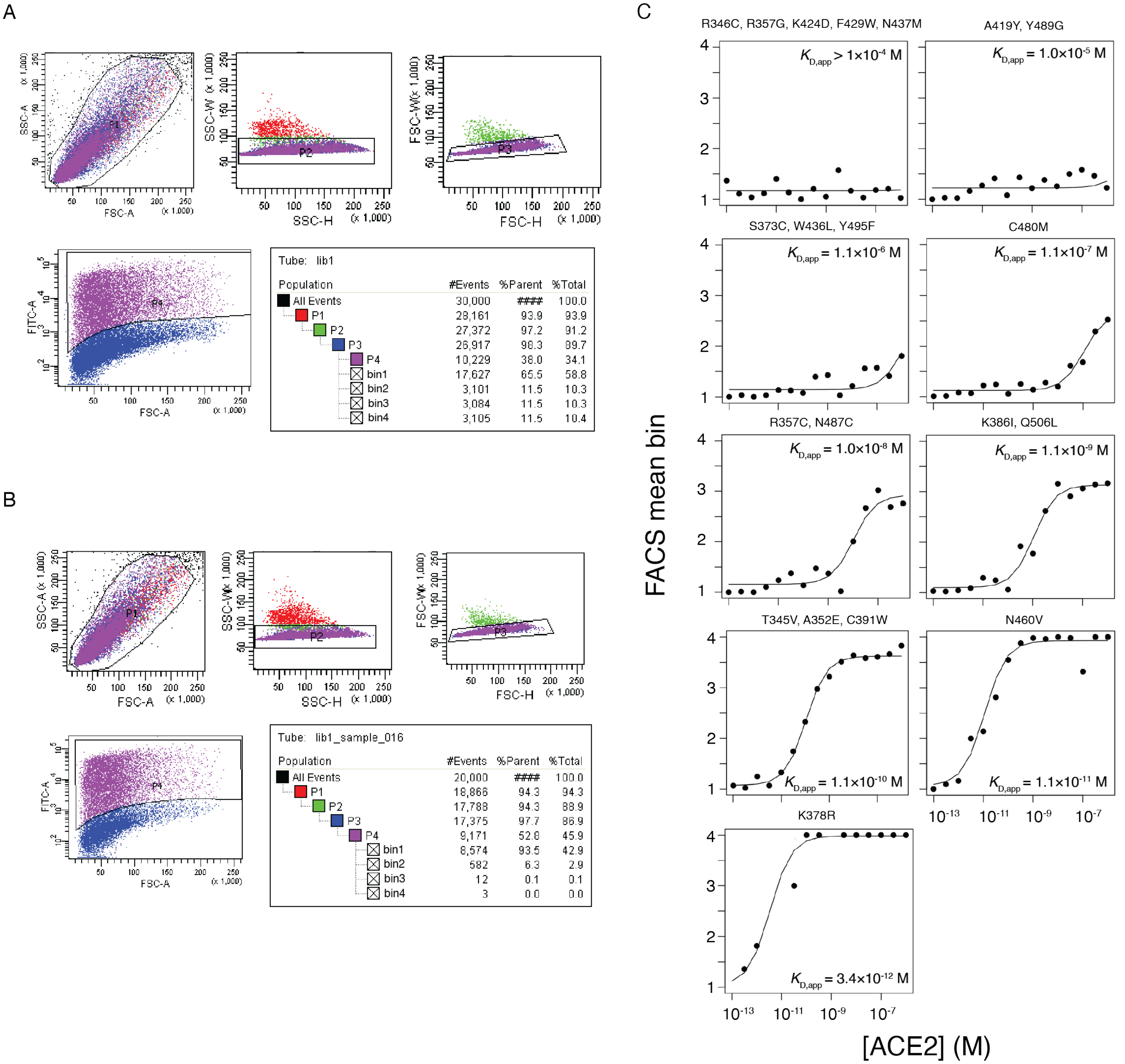

Figure S2. FACS-based determination of variant phenotypes. (A, B) Representative sorting gates used to select cells for for expression (A) and binding (B) FACS experiments. FSC and SSC gates select for single cells (P1-P3), and FITC labeling of an RBD C-terminal epitope tag defines RBD+ gates (P4), when necessary. Tables show the nested hierarchy of sort gates, with final bins 1-4 for expression and binding shown in Figure 2A and 2B, respectively. For (A), the P4 "RBD+" gate was used to enrich the library for expressing variants, which were grown up and re-induced for binding experiments as in (B). (C) Example variant-specific titration curves inferred from the deep mutational scanning experiment. Randomly sampled titration curves are illustrated across the range of fit $K_{D,app}$ binding constants, with variant genotype listed above each panel. Because curves that were fit with $K_{D,app}$ between 10^{-4} to 10^{-6} were virtually indistinguishable non-responsive curves, we truncated all $K_{D,app}$ measurements in this range to a censored $>10^{-6}$ M cutoff.

bioRxiv preprint doi: https://doi.org/10.1101/2020.06.17.157982. this version posted June 17, 2020. The copyright holder for this preprint (which was not certified by peer review) is the author/funder. It is made available under a CC-BY 4.0 International license.

Figure S3

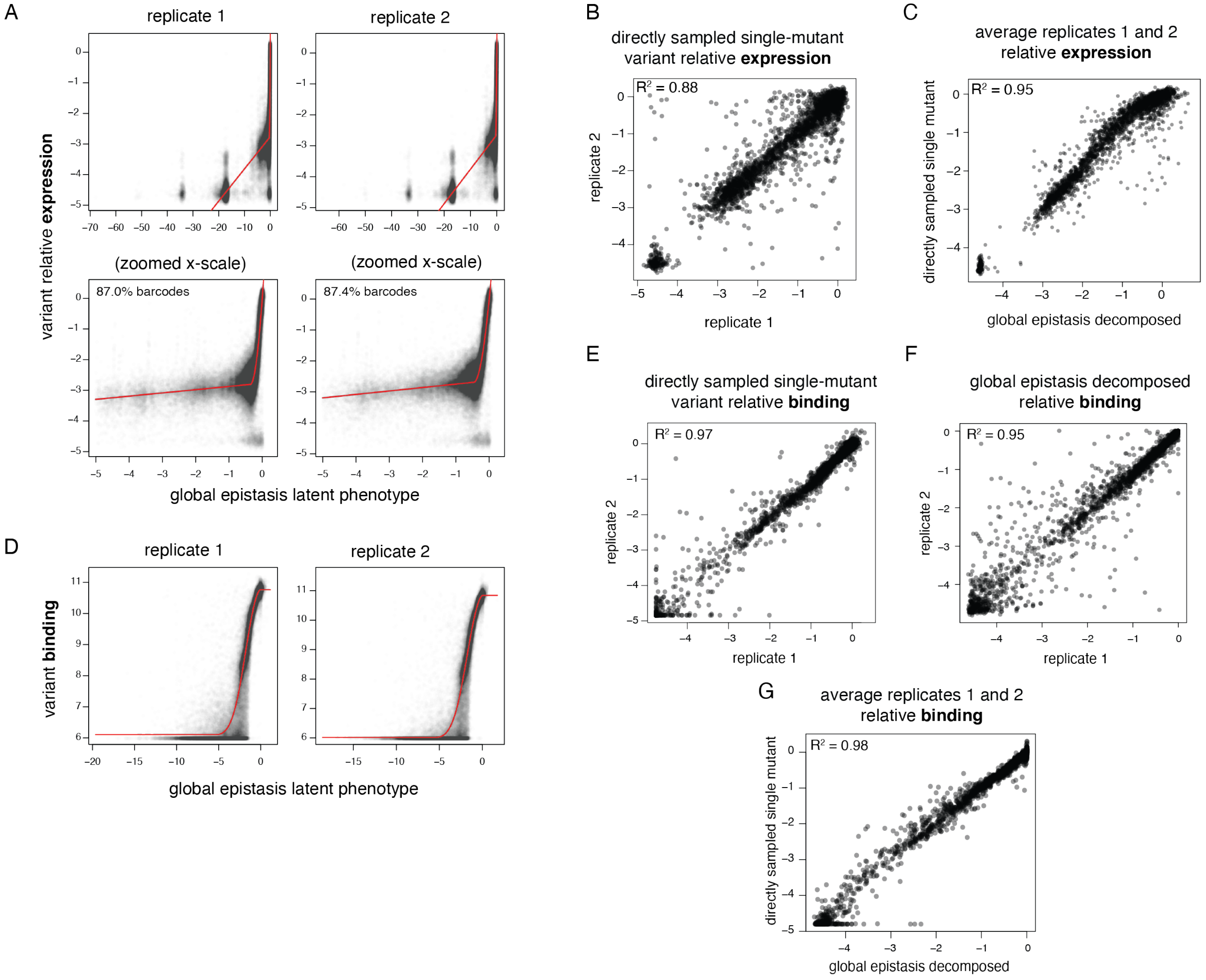

Figure S3. Global epistasis decomposition of single-mutant effects. Global epistasis models were fit to decompose single-mutant effects from variant backgrounds containing variable numbers of mutations. These models invoke an underlying latent scale on which mutations combine additively, which is linked to the experimental scale by a flexible nonlinear curve fit, which accounts for limits in dynamic range and other nonlinearities. See the Methods for more details. (A, D) Global epistasis fits. Plots illustrate, for each library variant, its experimentally determined phenotype for expression (A) or binding (D) versus its latent phenotype predicted by the global epistasis model. Red lines indicate the shape of the nonlinear curve fit. (B, E) Correlation in mutation effects on expression (B) and binding (E) between replicates, for mutations that were sampled directly as single mutants with no global epistasis decomposition. (F) Correlation in mutation effects on binding between replicates, for all global-epistasis-decomposed single-mutant effect terms on the observed phenotype scale. Equivalent plot for expression is Figure 2E. (C, G) Correlation in mutation effects on expression (C) and binding (G) averaged across replicates, for directly sampled single-mutant measurements versus global-epistasis-decomposed mutation effects. For expression, global epistasis averaging of single-mutant effects across all variants (Figure 2E) improved replicate correlations beyond the directly sampled measurements (B), so global-epistasis-decomposed values were used for all single-mutant terms. For binding, directly sampled single-mutant effects (E) were better correlated than the values decomposed from global epistasis models (F), so global epistasis models were used to interpolate single-mutant measurements only for mutations that were not observed on any directly-sampled single-mutant variant backgrounds.

bioRxiv preprint doi: https://doi.org/10.1101/2020.06.17.157982. this version posted June 17, 2020. The copyright holder for this preprint (which was not certified by peer review) is the author/funder. It is made available under a CC-BY 4.0 International license.

Figure S4

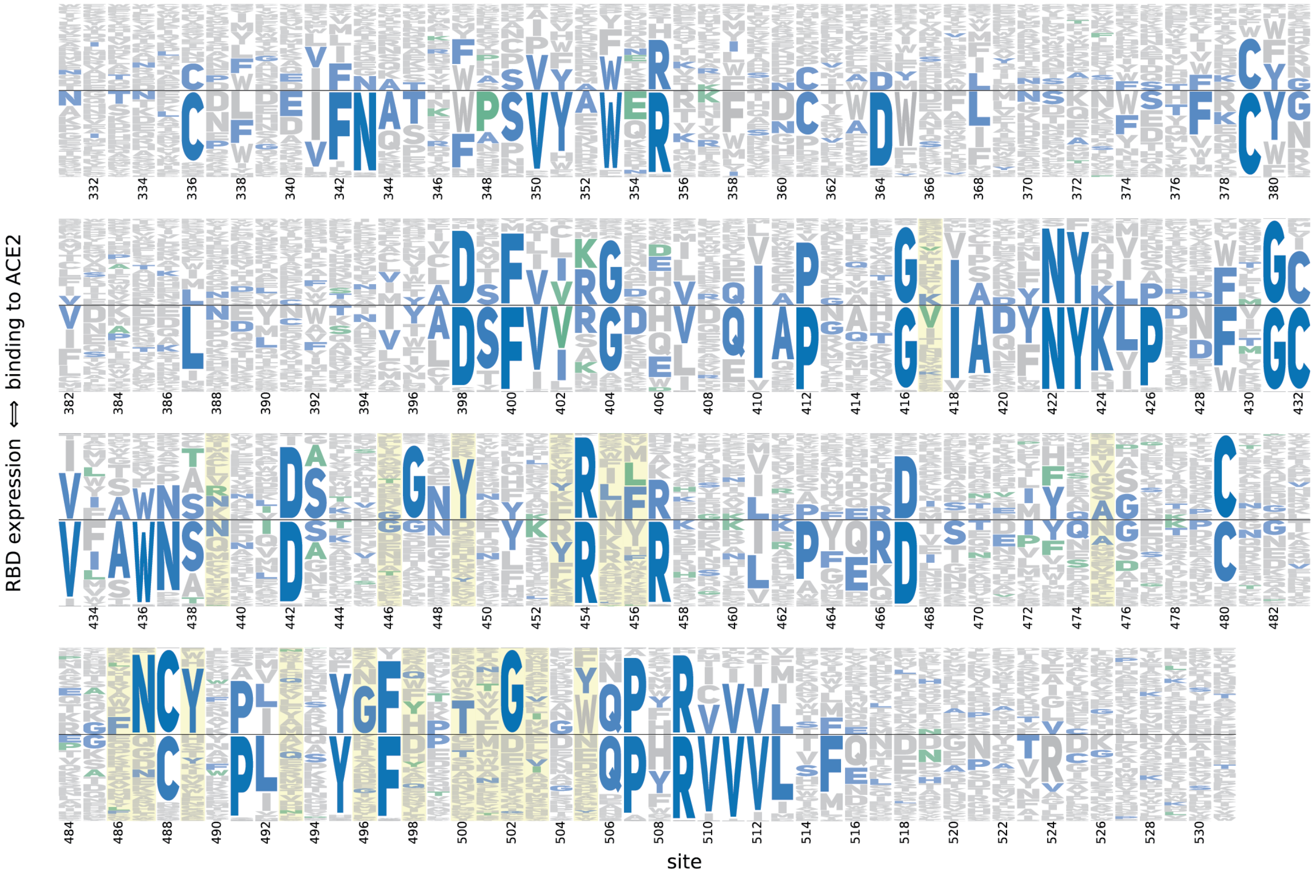

Figure S4 Logo plot representation of mutational effects on binding and expression. Letter height indicates preference of each site for individual amino acids with respect to ACE2 binding (height above the center line) or RBD expression (height below the center line). Blue letters indicate the unmutated SARS-CoV-2 amino acid, and, where applicable, green letters indicate differences found in SARS-CoV-1. Yellow highlights mark residues that contact ACE2 in the SARS-CoV-2 or SARS-CoV-1 crystal structures. See the Methods for details of how the amino-acid preferences are calculated from the experimental measurements.

bioRxiv preprint doi: https://doi.org/10.1101/2020.06.17.157982. this version posted June 17, 2020. The copyright holder for this preprint (which was not certified by peer review) is the author/funder. It is made available under a CC-BY 4.0 International license.

Figure S5.

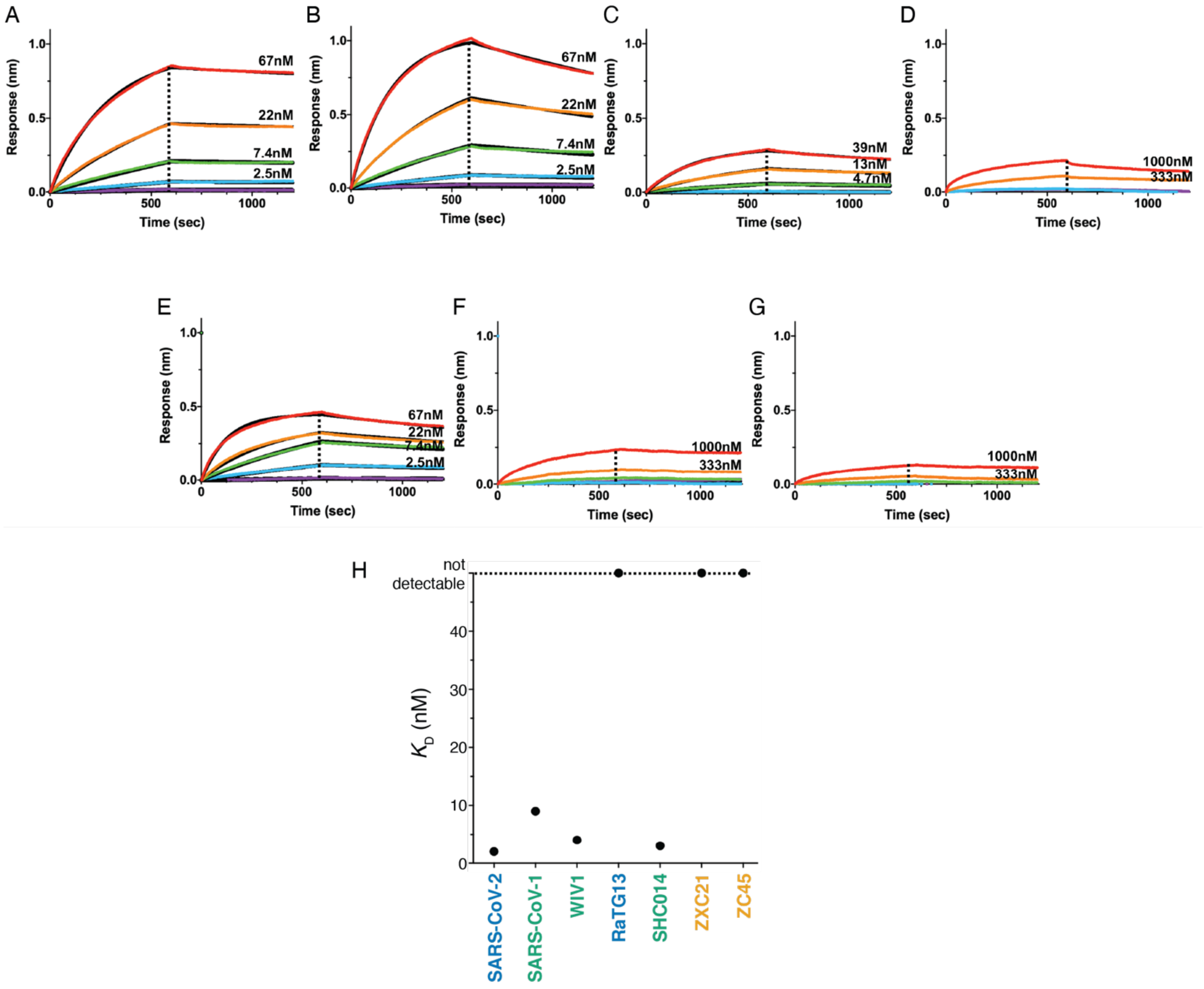

Figure S5. Human ACE2 binds to various sarbecovirus RBDs with distinct affinities. (A-G) Biolayer interferometry binding of various concentrations of monomeric ACE2 to the RBD of SARS-CoV-2 (A), SARS-CoV-1 (B), WIV1 (C), RaTG13 (D), SHC014 (E), ZXC21 (F) and ZC45 (G) immobilized at the surface of biosensors. Global fit curves are shown as black lines. The vertical dashed lines indicate the transition between association and dissociation phases. (H) Summary of K_D values determined from the shown BLI traces.

bioRxiv preprint doi: https://doi.org/10.1101/2020.06.17.157982. this version posted June 17, 2020. The copyright holder for this preprint (which was not certified by peer review) is the author/funder. It is made available under a CC-BY 4.0 International license.

Figure S6

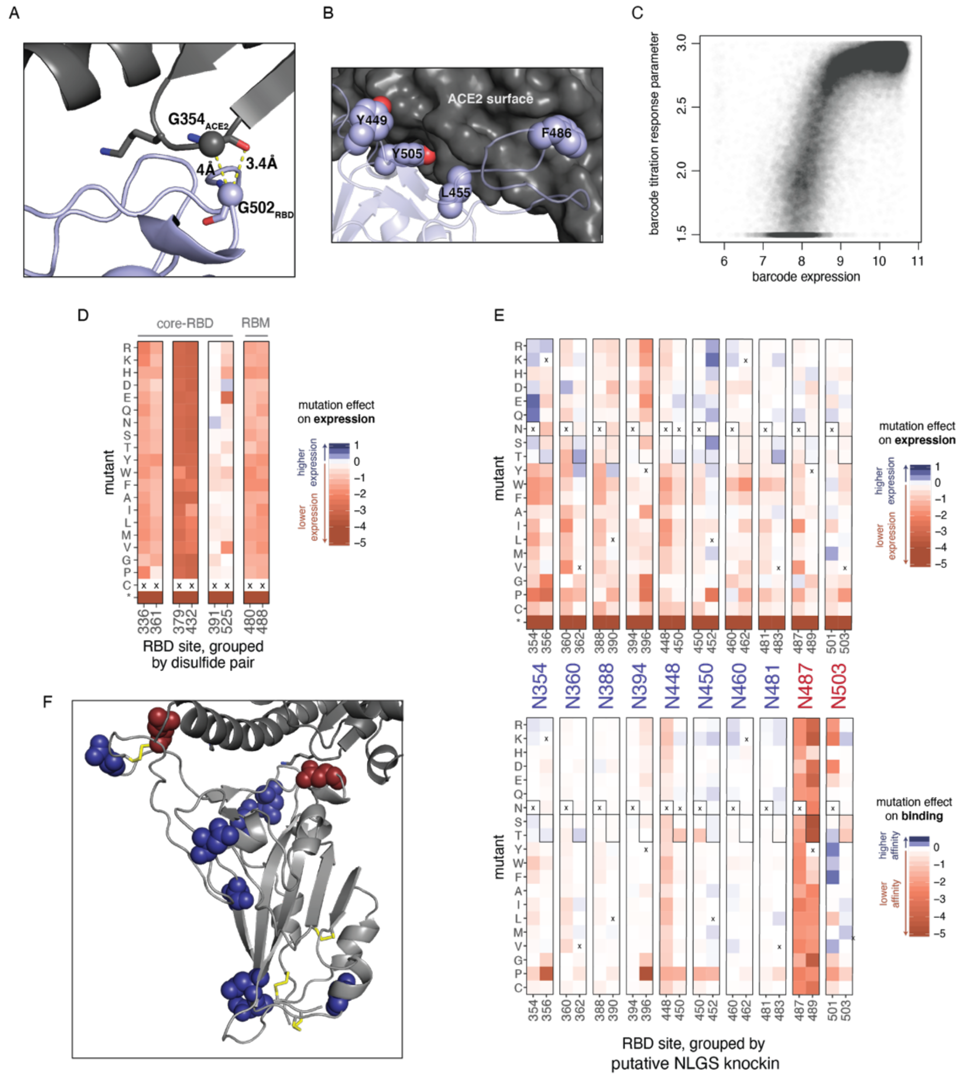

Figure S6. Additional structural analyses of mutation effects. (A, B) Structural depictions of sites exhibiting stability-binding tradeoffs. (A) RBD residue G502 requires small amino acid side chains for ACE2 binding (Figure 3B), consistent with its close proximity to $G354_{ACE2}$ in the bound structure. (B) Mutations to polar residues at positions Y449, L455, F486, and Y505 would enhance expression but reduce binding, consistent with specific geometric constraints imposed by the close packing of these residues at the ACE2 surface. (C) Relationship between barcode expression and titration response plateau parameters. The correlation between mutation effects on binding and expression in Figure 5C could emerge from trivial correlation between phenotypes (e.g. yeast with higher RBD surface expression can bind more ACE2). However, our multiple-concentration titration approach should in principle remove this trivial correlation (Adams et al., 2016), because each binding phenotype is determined from a self-referenced titration curve, for which the free plateau response parameter can vary to account for different levels of saturated binding due to RBD expression (see Figure S2C). Consistent with this premise, the response parameter from the titration fit for each library variant correlates with its expression phenotype. (D) Mutation effects on expression at disulfide cysteine residues. Details as in figure 5E. (E) Effects of putative N-linked glycosylation site (NLGS) knock-in mutations. Heatmap details as in Figure 5F. There are 10 surface-exposed asparagines for which RBD expression is unaffected or enhanced (top) when an NLGS motif is introduced via mutations to S or T at the i+2 site; for eight of these putative NLGS knock-ins (blue labels), the putative glycan is also tolerated for ACE2 binding (bottom), but for two (red labels), introduction of the NLGS motif is not tolerated for ACE2 binding. (F) Mapping of these ten asparagines to the RBD structure illustrates that these two binding-constrained asparagines (red) cluster to the ACE2 interface.

bioRxiv preprint doi: https://doi.org/10.1101/2020.06.17.157982. this version posted June 17, 2020. The copyright holder for this preprint (which was not certified by peer review) is the author/funder. It is made available under a CC-BY 4.0 International license.

Figure S7

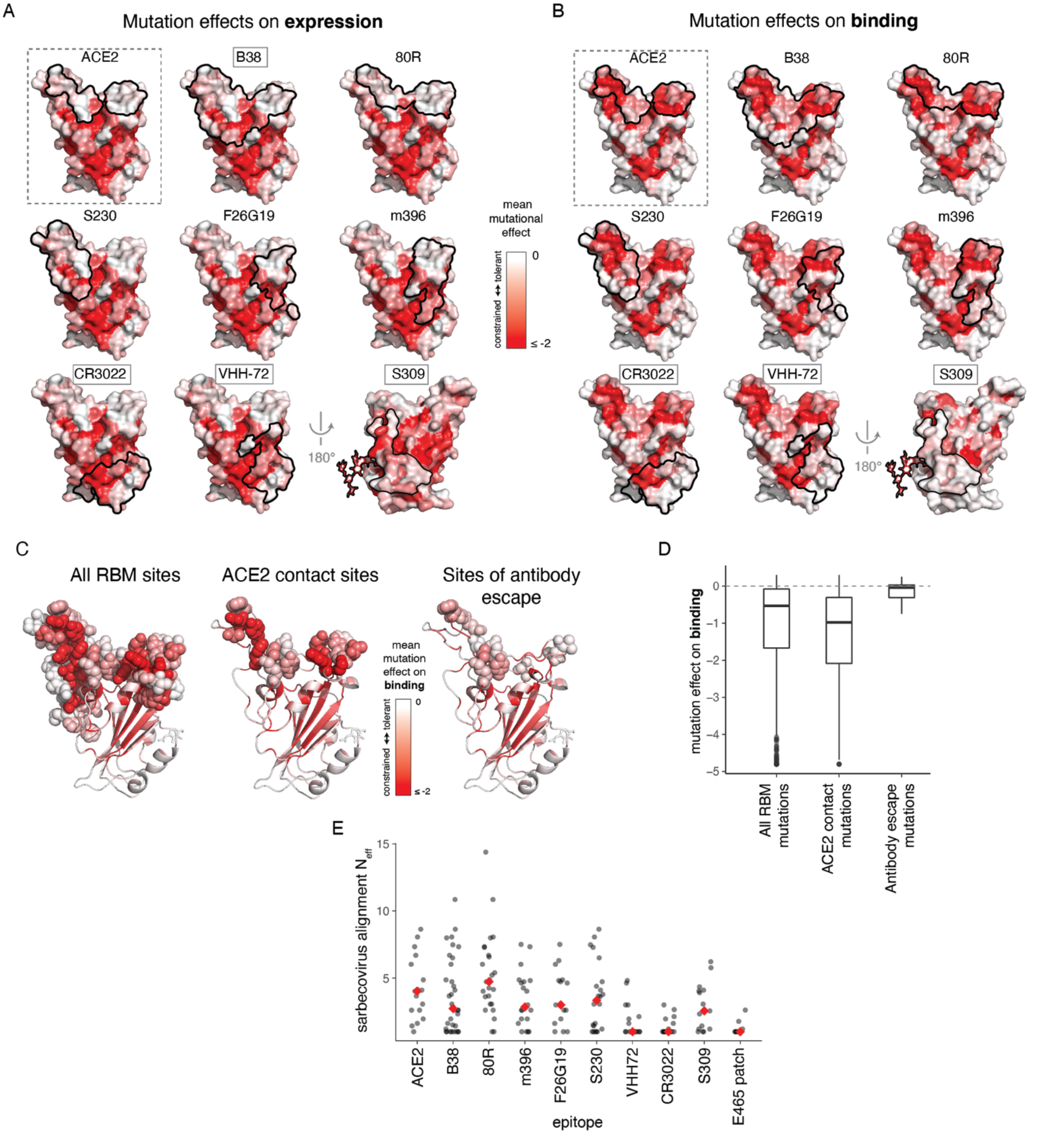

Figure S7. Mutational and evolutionary constraint of antibody epitopes. (A, B) Surface representations of antibody epitopes colored by mutational effects on expression (A) and binding (B). Representations as described in Figure 7A. (C,D) Mutational constraint and observed antibody escape mutations. Baum et al. (Baum et al., 2020) selected SARS-CoV-2 escape mutations from RBD-directed antibodies. We compare the average mutational tolerance of the sites at which these escape mutations accrue (C), and the effects of the specific escape mutations themselves (D) to all RBM and ACE2-contact sites/mutations. The antibody escape involved mutations that were better tolerated than typical mutations in the RBM or ACE2-binding interface. (E) Evolutionary diversity in antibody epitopes and our newly described E465-centered surface patch among the sarbecoviruses in Figure 1A. Diversity is summarized as the effective number of amino acids (N_{eff}), which scales from 1 for a site that is invariant, to 20 for a site in which all amino acids are at equal frequency.

bioRxiv preprint doi: https://doi.org/10.1101/2020.06.17.157982. this version posted June 17, 2020. The copyright holder for this preprint (which was not certified by peer review) is the author/funder. It is made available under a CC-BY 4.0 International license.

Figure S8

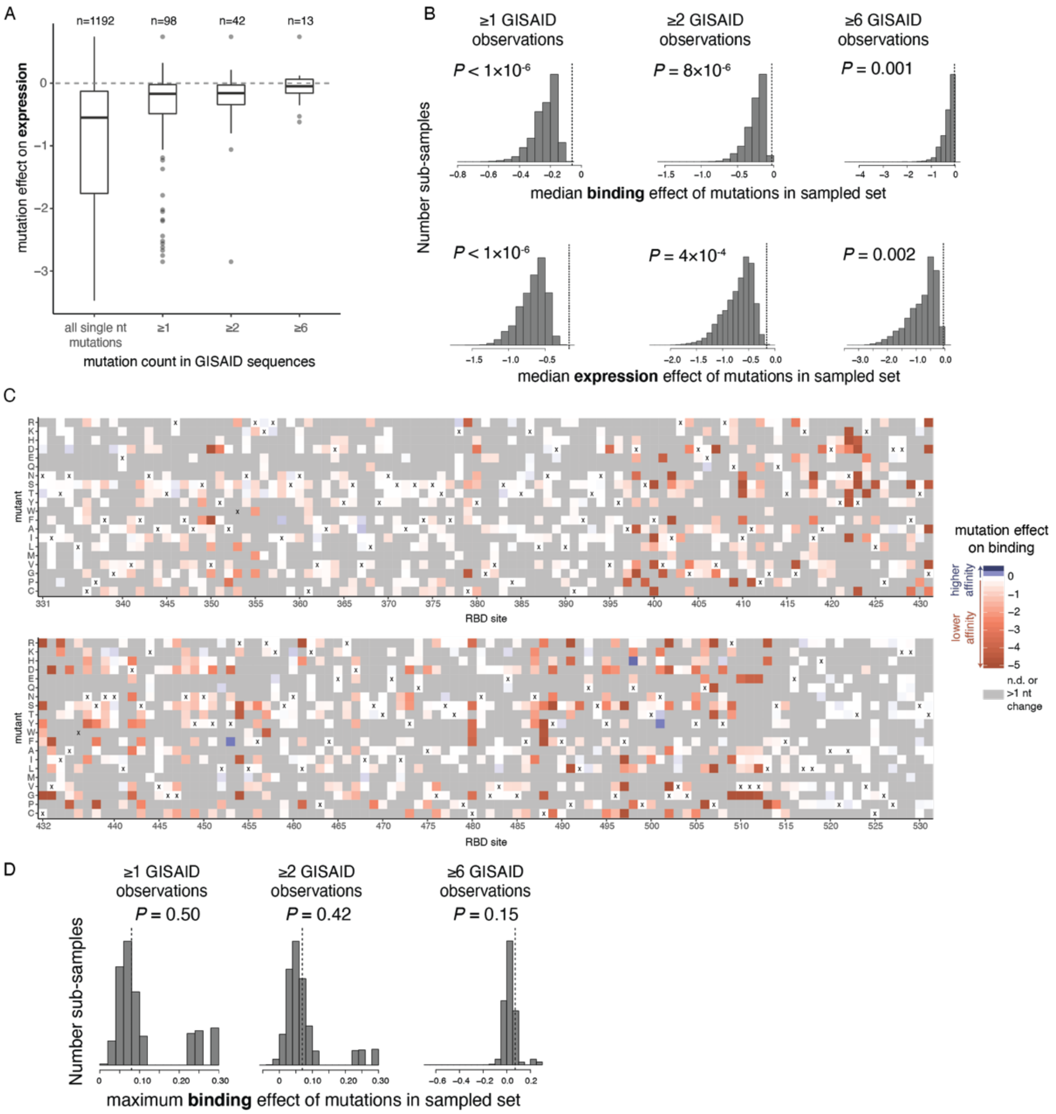

Figure S8. Genetic variation and selection in SARS-CoV-2. (A) Distribution of expression effects of mutations observed among circulating SARS-CoV-2 isolates. Details as in Figure 8A. (B) Permutation tests indicating the action of purifying selection on binding (top) and expression (bottom) among circulating SARS-CoV-2 mutations. For each threshold of GISAID observation counts, 1 million random sub-samples of single-nucleotide-accessible amino acid changes were generated at the same sample size as the true mutation set (n=98, 42, and 13 for the ≥1, ≥2, and ≥6 thresholds). A *P*-value was determined as the fraction of sub-samples with median mutational effect on binding or expression equal to or greater than that of the actual GISAID mutation set (dashed vertical line). The observation that the set of mutations observed in GISAID have a more favorable median mutational effect on binding and expression than randomly sampled mutations indicates the action of purifying selection for ACE2 binding and RBD stability. (C) Heatmaps depicting effects of mutations on ACE2 binding, indicating only those mutations that are accessible via single-nucleotide mutation from the SARS-CoV-2 Wuhan-Hu-1 isolate gene sequence. Amino-acid mutations that require more than one nucleotide change are in gray. (D) Permutation tests for positive selection for enhanced ACE2 affinity. Random sub-samples were generated as in (B), and the maximum affinity-enhancing effect of mutations in each sub-sample was compared to that in the actual GISAID mutation set. A *P*-value was determined as the fraction of sub-samples with a maximum effect on binding equal to or greater than in the actual GISAID mutation set (vertical dashed line). We do not see evidence for selection for enhanced ACE2 binding, as randomly sampled mutations generally contain mutations with stronger affinity-enhancing effects than observed in the GISAID mutation set.

bioRxiv preprint doi: https://doi.org/10.1101/2020.06.17.157982. this version posted June 17, 2020. The copyright holder for this preprint (which was not certified by peer review) is the author/funder. It is made available under a CC-BY 4.0 International license.

List of Supplemental Files:

- Supplemental File 1: CSV containing binding and expression measurements for RBD homologs spiked into the deep mutational scanning libraries
- Supplemental File 2: html file of the interactive heatmap provided via url in the main text
- Supplemental File 3: CSV file containing all single-mutant deep mutational scanning measurements from our duplicate experiments
